# Defective Protein Prenylation in a Spectrum of Patients With Mevalonate Kinase Deficiency

**DOI:** 10.3389/fimmu.2019.01900

**Published:** 2019-08-14

**Authors:** Marcia A. Munoz, Julie Jurczyluk, Anna Simon, Pravin Hissaria, Rob J. W. Arts, David Coman, Christina Boros, Sam Mehr, Michael J. Rogers

**Affiliations:** ^1^Bone Biology, Garvan Institute of Medical Research, Sydney & St Vincent's Clinical School, UNSW Sydney, Sydney, NSW, Australia; ^2^Department of Internal Medicine, Radboudumc Expertise Centre for Immunodeficiency and Autoinflammation, Radboud University Medical Centre, Nijmegen, Netherlands; ^3^Royal Adelaide Hospital, Adelaide, SA, Australia; ^4^Queensland Children's Hospital, Brisbane, QLD, Australia; ^5^School of Medicine, University of Queensland, Brisbane, QLD, Australia; ^6^School of Medicine, Griffith University, Brisbane, QLD, Australia; ^7^Department of Rheumatology, Women's and Children's Hospital, University of Adelaide Discipline of Paediatrics, Adelaide, SA, Australia; ^8^Department of Allergy and Immunology, Royal Children's Hospital, Melbourne, VIC, Australia

**Keywords:** HIDS, mevalonate kinase, prenylation, Rab GTPase, Rap1, autoinflammation

## Abstract

The rare autoinflammatory disease mevalonate kinase deficiency (MKD, which includes HIDS and mevalonic aciduria) is caused by recessive, pathogenic variants in the *MVK* gene encoding mevalonate kinase. Deficiency of this enzyme decreases the synthesis of isoprenoid lipids and thus prevents the normal post-translational prenylation of small GTPase proteins, which then accumulate in their unprenylated form. We recently optimized a sensitive assay capable of detecting unprenylated Rab GTPase proteins in peripheral blood mononuclear cells (PBMCs) and showed that this assay distinguished MKD from other autoinflammatory diseases. We have now analyzed PBMCs from an additional six patients with genetically-confirmed MKD (with different compound heterozygous *MVK* genotypes), and compared these with PBMCs from three healthy volunteers and four unaffected control individuals heterozygous for the commonest pathogenic variant, *MVK*^*V*377*I*^. We detected a clear accumulation of unprenylated Rab proteins, as well as unprenylated Rap1A by western blotting, in all six genetically-confirmed MKD patients compared to heterozygous controls and healthy volunteers. Furthermore, in the three subjects for whom measurements of residual mevalonate kinase activity was available, enzymatic activity inversely correlated with the extent of the defect in protein prenylation. Finally, a heterozygous *MVK*^*V*377*I*^ patient presenting with autoinflammatory symptoms did not have defective prenylation, indicating a different cause of disease. These findings support the notion that the extent of loss of enzyme function caused by biallelic *MVK* variants determines the severity of defective protein prenylation, and the accumulation of unprenylated proteins in PBMCs may be a sensitive and consistent biomarker that could be used to aid, or help rule out, diagnosis of MKD.

## Introduction

Mevalonate kinase deficiency (MKD) is a rare, autosomal recessive, autoinflammatory disorder caused by pathogenic variants in the *MVK* gene (OMIM 251170) encoding the metabolic enzyme mevalonate kinase (MK) (1, 2). The symptoms of MKD usually appear in early childhood. The milder end of the clinical spectrum is characterized by recurrent episodes of high fever with abdominal pain, lymphadenopathy, vomiting, diarrhea and gastrointestinal inflammation, rashes, and debilitating joint pain. Many patients have high serum concentrations of IgD and IgA (hence the alternative name hyperimmunoglobulinaemia D and periodic fever syndrome, or HIDS; OMIM 260920) (3, 4). The more severe cases of MKD (known as mevalonic aciduria; OMIM 610377) also present with neurological and developmental abnormalities, and risk of premature death from complications including pulmonary failure (5).

Decreased activity of the MK enzyme (ATP:mevalonate 5-phosphotransferase; EC 2.7.1.36) leads to intracellular depletion of lipid precursors that are essential for the post-translational prenylation of proteins, particularly small GTPase proteins such as those of the Rab family of GTPases (6). It was therefore proposed that protein prenylation is defective in MKD and could be the underlying cause of inflammation (7–9), however direct evidence for defective prenylation in patient samples has been lacking. Furthermore, diagnostic biochemical features of MKD are not always reliable: elevated urinary mevalonic acid levels are only detected during a fever episode and raised serum IgD or IgA is not present in all patients (10). Genetic analysis to detect homozygous or compound heterozygous variants in *MVK* that are pathogenic or likely pathogenic offers the most reliable diagnosis, although this does not indicate whether previously unreported variants actually give rise to functionally deficient MK enzyme. We recently developed a method to detect subtle changes in the prenylation of Rab GTPase proteins (11, 12) and showed in three patients for the first time that prenylation of Rab proteins, as well as Rap1A, is indeed defective in peripheral blood mononuclear cells (PBMCs) from MKD and could be a biomarker that distinguishes this disease from other autoinflammatory disorders with similar clinical features (13). However, it remains to be confirmed whether this assay consistently identifies MKD in patients with diverse *MVK* genotypes. In this study, we analyzed an additional six patients with genetically-confirmed MKD and different compound heterozygous *MVK* genotypes, four healthy individuals that were heterozygous for the commonest pathogenic variant *MVK*^*V*377*I*^, one patient with autoinflammatory disease but heterozygous for *MVK*^*V*377*I*^, and three healthy controls.

## Methods

### Participants

The study was approved by the Sydney Children's Hospitals Network Human Research Ethics Committee (HREC/18/SCHN/403) and participant details are summarized in Supplementary Table 1. Healthy control volunteers were adult Caucasian males. Patients P1–P4 were adults, randomly selected from a well-described cohort in Nijmegen, the Netherlands (14). All 4 individuals were compound heterozygous for biallelic pathogenic or likely pathogenic variants in *MVK*, having the most common pathogenic variant *MVK*^*V*377*I*^ on one allele and a point mutation (H20P, I268T, G326R) or undetermined insertion/deletion on the other allele (genetic details summarized in Supplementary Table 2). P1 *(MVK*^*V*377*I*/*H*20*P*^*)*, an adult female, originally presented with classical HIDS phenotype (age of onset 2 months, inflammatory attacks lasting 5–6 days, every 4–6 weeks) and was receiving long-term etanercept treatment at the time of blood sampling. P2 (*MVK*^*V*377*I*/*I*268*T*^), an adult male, originally presented with typical symptoms of inflammatory episodes of headache, abdominal pain, arthritis, skin lesions, lymphadenopathy, hepatomegaly, and splenomegaly from 6 months of age. He was receiving canakinumab treatment at the time of blood sampling. P3 (*MVK*^*V*377*I*/*G*326*R*^) was an adult female who showed very classical HIDS presentation with disease onset at 3 months of age, fever episodes lasting 5 days, every 2–3 weeks. She was on canakinumab treatment at time of blood draw. P4 (*MVK*^*V*377*I*/*indel*^*)*, an adult male, presented at 6 years of age with episodes of arthralgia/myalgia, skin lesions, arthritis, lymphadenopathy, and vomiting lasting 7–10 days every 8 weeks. He was not on any anti-inflammatory treatment at the time of blood sampling.

Patients P5, P6, and P7, identified in Australia, had a clinical history of autoinflammatory disease. P5, a young adult male, had periodic episodes of fever, polyarthralgias, abdominal pain, and diarrhea every month from 6 months of age. He had no family history of note and, at 15 years of age, was diagnosed as compound heterozygous for *MVK* variants (*MVK*^*V*377*I*^ and a novel, likely pathogenic duplication/insertion in exon 5 leading to insertion of 2 amino acids; Supplementary Table 2). His symptomatology improved with age and symptoms became less frequent. He had moderate response to anti-IL-1 therapy, which was given for 2 years, and was being managed with on-demand NSAIDs. P6, an adult female, also carried the pathogenic *MVK*^*V*377*I*^ variant sequence on one allele, but lacked genetic diagnosis of a specific autoinflammatory disease. She had episodic large joint arthritis, intermittent macular rash, with fevers, lymphadenopathy, and mild splenomegaly since 15 years of age. She did not respond to multiple disease-modifying anti-rheumatic medications including low dose steroids but eventually had complete response to anti IL-1 therapy (anakinra). She had a daughter who was diagnosed with juvenile idiopathic arthritis at 4 years of age. P7 was the daughter of non-consanguineous parents of European ancestry. At 5 years of age, she diagnosed with mild dystonic cerebral palsy. During the next 2 years, she developed a periodic fever pattern cycling on a 2–4 weekly basis, with temperatures of 39–40°C associated with significant elevations in inflammatory markers, e.g., CRP and ESR. At 6 years of age, a diagnosis of a monogenic periodic fever syndrome was entertained and a short course of 1 mg/kg oral prednisolone given during a febrile episode resulted in a complete ablation of the dystonic posturing and neurological symptoms. Subsequent investigations demonstrated persistently elevated IgD titres (721–803 mg/L RR < 159) and mild elevation of urinary mevalonic acid. Whole exome sequencing revealed biallelic, pathogenic *MVK* variants (*MVK*^*V*377*I*/*Y*114Ifs^*^71^) inherited from each heterozygous parent (Supplementary Table 2) and a maternally inherited variant of the *TOR1A* gene (NM_000113 c.962C>T; p.Thr321Met). The mother had no clinical features of dystonia and had a normal neurological examination.

### *In vitro* Prenylation (IVP) Assay

All subjects gave written informed consent in accordance with the Declaration of Helsinki, prior to obtaining fresh peripheral blood samples. All samples were drawn whilst individuals were without inflammatory flares. PBMCs were isolated by centrifugation over Ficoll; cells from P1, P2, P3, P4 collected in Nijmegen, the Netherlands, were frozen in fetal calf serum with 10% DMSO prior to shipping to Garvan Institute, Australia, on dry ice whereas PBMCs from P5, P6, P7 were isolated by centrifugation over Ficoll then washed twice with PBS, snap-frozen as cell pellets and stored at −80°C.

Frozen pellets of viable cells that were thawed and centrifuged (P1–P4), or snap-frozen cell pellets (P5–P7), were lysed by sonication in ~100 μl prenylation buffer (50 mM HEPES pH 7.2, 50 mM NaCl, 2 mM MgCl_2_, 100 μM GDP, 1x Roche complete EDTA-free protease inhibitor cocktail). Protein was quantified using a BCA assay (Pierce) then *in vitro* prenylation assays to detect unprenylated Rab GTPases were performed as previously described (11). Briefly, DTT was added to 50 μg cell lysate to a final concentration of 2 mM, with final concentrations of 2 μM Rab GGTase, 2 μM recombinant *Danio rerio* Rab escort protein-1 (REP-1), 0.5 μM B-GPP in a total volume of ~50 μL and reactions incubated for 5 h at room temperature. *In vitro* prenylated (i.e., biotinylated) Rab proteins were detected on PVDF blots using streptavidin-680RD (LiCOR). A narrow doublet (often appearing as a broad singlet) of endogenous biotinylated 75 kDa protein was used as a sample loading control. Blots were also analyzed for unprenylated Rap1A by western blotting using goat anti-Rap1A (sc-1482) as previously described (11).

## Results

**Figure 1:**
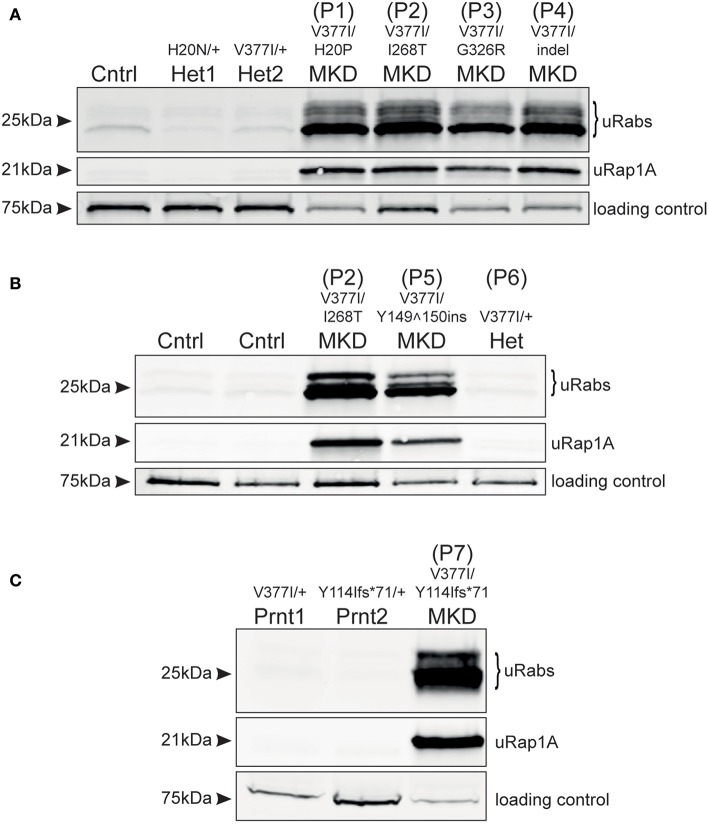
Defective protein prenylation in PBMCs is detected consistently in patients with MKD. **(A)** PBMCs from four adult, compound heterozygous MKD patients (P1–P4) have clear accumulation of unprenylated Rab GTPases (uRabs) and unprenylated Rap1A (uRap1) compared to a healthy control (Cntrl) and 2 heterozygous individuals (Het1,2). **(B)** PBMCs from a young adult patient with MKD (P5) show accumulation of unprenylated Rab and Rap1A GTPases compared to 2 healthy controls (Cntrl) and a heterozygous (Het) individual (P6) with undiagnosed inflammatory disease. Patient P2 is also shown alongside for comparison. **(C)** A child (P7) with MKD shows a severe defective in Rab and Rap1A prenylation in PBMCs, compared to both unaffected, heterozygous parents (Prnt1,2). Seventy-five kilodalton endogenous biotinylated protein was used as a loading control in all of the blots.

Analysis of frozen, viable PBMCs obtained from 4 MKD patients in the Netherlands (P1–P4) demonstrated a clear accumulation of unprenylated Rab GTPases and unprenylated Rap1A (Figure 1A), compared to PBMCs from a healthy control and two unaffected heterozygous carriers of *MVK*^*H*20*N*^ and *MVK*^*V*377*I*^ variants [the latter described previously, (13)]. Patients P1, P2, and P4, with *MVK*^*V*377*I*/*H*20*P*^, *MVK*^*V*377*I*/*I*268*T*^, and *MVK*^*V*377*I*/*indel*^ genotypes, respectively, appeared to have a more severe defect in prenylation than patient P3, with the *MVK*^*V*377*I*/*G*326*R*^ genotype. MK enzyme activity has previously been determined in cultured lymphocytes from patients P1, P3, and P4 (14) (Supplementary Table 1). P1 and P4 showed a very similar defect in prenylation (Figure 1A) and both had 8.9% of normal MK activity, whereas P3 with a higher residual MK enzyme activity (16.4%) had a milder prenylation defect (Figure 1A).

Analysis of PBMCs from patient P5 (*MVK*^*V*377*I*/*Y*149^150*ins*^^) revealed a clear defect in Rab and Rap1A prenylation (Figure 1B; note that P5 is shown alongside P2 for comparison with Figure 1A). PBMCs from the heterozygous individual P6 (*MVK*^*V*377*I*/+^) did not have a prenylation defect and appeared the same as PBMCs from two healthy control volunteers (Figure 1B). PBMCs from P7 (*MVK*^*V*377*I*/Y114Ifs^*^71^) had a severe defect in Rab and Rap1A prenylation (Figure 1C) that was completely absent in both parents heterozygous for these *MVK* variants.

## Discussion

We recently proposed that MKD can be identified and distinguished from other autoinflammatory disorders by the defect in protein prenylation, and that this defect can be detected using an *in vitro* prenylation assay (11) to analyze PBMC patient samples (12, 13). We now show, with 6 additional, genetically-confirmed MKD patients, that the assay accurately revealed the accumulation of unprenylated proteins only in the PBMCs from 6 patients carrying pathogenic/likely pathogenic MKD variants on both alleles (P1, P2, P3, P4, P5, and P7—see Supplementary Table 2). Furthermore, the underlying defect in protein prenylation was still apparent in PBMCs of 3 patients despite anti-inflammatory treatment (etanercept or canakinumab) at the time of blood sampling. Interestingly, heterozygous *MVK*^*V*377*I*/+^ individual P6 did not have defective protein prenylation even though she presented with autoinflammatory disease. This is entirely consistent with the recessive nature of pathogenic *MVK* variants (3, 4) and her symptoms were therefore most likely due to a different disorder.

It remains unclear how the severity of mutations in *MVK* correlate with loss of MK enzyme activity and hence the extent of the defect in protein prenylation. Point mutations that alter amino acid positions 8–35 and 234–338 (which includes the mutant *MVK* alleles in patients P1, P2, P3) affect regions adjacent to the active site cleft or dimerization interface of MK and appear to have the most deleterious effect on enzyme folding or stability (15). Pathogenic variants affecting other regions, such as the Valine to Isoleucine substitution at position 377 (*MVK*^*V*377*I*^)—the commonest pathogenic variant in MKD (3, 16, 17)—may render the enzyme temperature-sensitive (4, 18, 19). We previously found a very mild prenylation defect in a patient homozygous for *MVK*^*V*377*I*^ (13), a genotype associated with mild or even absent clinical features (20). In the current study we found that the prenylation defect appeared more pronounced in patients with lower residual MK activity (P1, P4), and milder in a patient (P3) with higher residual MK activity. Furthermore, patient P7 showed a very profound defect in protein prenylation and presented with severe clinical symptoms. Hence, at least in this small group of patients, the extent of the defect in protein prenylation in PBMCs (as detected in our assay) seems to correlate with how severely the mutations affect MK enzyme activity. Because of the rarity of patients with mevalonic aciduria (the severest form of MKD), we have not yet been able to analyse PBMCs from any of these individuals. However, because MK enzyme activity is usually undetectable in mevalonic aciduria patients we would predict that the defect in protein prenylation in these individuals would be even more dramatic than in HIDS (the milder form of MKD). Analysis of a larger cohort of patients is clearly needed to better understand the relationship between the severity of pathogenic *MVK* variants, defective prenylation and clinical symptoms in MKD. Nevertheless, our studies show that the *in vitro* prenylation assay described here appears to be a sensitive and accurate method that could aid, or help rule out, a specific diagnosis of MKD in patients presenting with autoinflammatory disease. Finally, it is worth noting that the analysis of patients P1, P2, P3, and P4 (Figure 1A) was performed on frozen, viable PBMCs shipped on dry ice to Australia by courier from the Netherlands, whilst the analysis of patients P5, P6, and P7 (Figures 1B,C) was performed on freshly-isolated and snap-frozen cells stored at −80°C. The results demonstrate that the *in vitro* prenylation assay was effective at detecting the accumulation of unprenylated proteins in both sets of PBMC samples. Hence, PBMC samples can be collected, stored and shipped to our institute or elsewhere for analysis without compromising the accuracy of the results.

## Data Availability

The raw data supporting the conclusions of this manuscript will be made available by the authors, without undue reservation, to any qualified researcher.

## Ethics Statement

All subjects gave written informed consent in accordance with the Declaration of Helsinki. The protocol was approved by the Sydney Children's Hospitals Network Human Research Ethics Committee (HREC/18/SCHN/403).

## Author Contributions

MM and MR designed the study, analyzed the data and wrote the manuscript. JJ performed the assays and contributed to the manuscript. AS, PH, RA, DC, CB, and SM recruited participants and contributed to the manuscript.

### Conflict of Interest Statement

The authors declare that the research was conducted in the absence of any commercial or financial relationships that could be construed as a potential conflict of interest.

## Abbreviations

GGTase: geranylgeranyltransferase
MKD: mevalonate kinase deficiency
MK: mevalonate kinase (protein)
*MVK*: mevalonate kinase (gene)
HIDS: Hyper IgD and periodic fever syndrome.
