# Supplementary material for: Defective Protein Prenylation in a Spectrum of Patients With Mevalonate Kinase Deficiency
Source: Front Immunol. 2019 Aug 14;10:1900. doi: 10.3389/fimmu.2019.01900 (PMC6702261; doi:10.3389/fimmu.2019.01900)
Supplement: Supplementary file 1 [file Table_1.DOCX]

**Supplementary Table 1.**

Details of participants in the study with compound heterozygous *MVK* sequence variants (P1-P5, P7) or heterozygous for a *MVK* variant (P6, Het1, Het2, Parent1, Parent2). All participants were of Caucasian/European ancestry. Healthy control volunteers in Fig. 1 were adult male Caucasians.

| **Participant number** | **Sex** | **Age** | **Clinical features** | **Medication at sampling time** |
| --- | --- | --- | --- | --- |
| P1 | F | adult | inflammatory attacks lasting 5-6 days every 4-6 weeks, from 2 months of age | etanercept |
| P2 | M | adult | headache, abdominal pain, arthritis, skin lesions, lymphadenopathy, hepatomegaly and splenomegaly, from 6 months of age | canakinumab |
| P3 | F | adult | fever episodes lasting 5 days every 2-3 weeks, from 3 months of age | canakinumab |
| P4 | M | adult | arthralgia/myalgia, skin lesions, arthritis, lymphadenopathy and vomiting lasting 7-10 days every 8 weeks, from age 6 | none |
| P5 | M | adult | periodic episodes of fever, polyarthralgias, abdominal pain and diarrhoea every month from 6 months of age | NSAID |
| P6 | F | adult | episodic large joint arthritis, intermittent macular rash, with fevers, lymphadenopathy and mild splenomegaly from age 15 | none |
| P7 | F | child | mild dystonic cerebral palsy, elevated serum IgD and urinary mevalonic acid, periodic fevers every 2-4 weeks, from age 5 | none |
| Het1 | M | adult | - | - |
| Het2 | F | adult | - | - |
| Parent1 | M | adult | - | - |
| Parent2 | F | adult | - | - |
