# Supplementary material for: Defective Protein Prenylation in a Spectrum of Patients With Mevalonate Kinase Deficiency
Source: Front Immunol. 2019 Aug 14;10:1900. doi: 10.3389/fimmu.2019.01900 (PMC6702261; doi:10.3389/fimmu.2019.01900)
Supplement: Supplementary file 2 [file Table_2.DOCX]

**Supplementary Table 2.**

*MVK* genotypes of participants analysed in the study. *MVK* genotypes show nucleotide and amino acid changes from the standard reference sequence (NM_000431). Compound heterozygous individuals P1, P2, P3, P4, P5, P7 had biallelic, pathogenic(^#^)/likely pathogenic(^##^) variants in *MVK*; Heterozygous individuals P6, Het1, Het2, Prnt1, Prnt2 had a pathogenic(^#^)/likely pathogenic(^##^) variant in a single allele of *MVK*. Values of residual MK activity are from Cuisset *et al*, 2001 [14]; nd = not determined.

| **Participant number** | ***MVK* variant, allele 1** | ***MVK* variant, allele 2** | **Residual MK activity** |
| --- | --- | --- | --- |
| P1 | c.1129G>A (p.Val377Ile) ^#^ | c.59A>C (p.His20Pro) ^#^ | 8.9% |
| P2 | c.1129G>A (p.Val377Ile) ^#^ | c.803T>C (p.Ile268Thr) ^#^ | nd |
| P3 | c.1129G>A (p.Val377Ile) ^#^ | c.976G>A (p.Gly326Arg)^##^ | 16.4% |
| P4 | c.1129G>A (p.Val377Ile) ^#^ | undetermined insertion/deletion^##^ | 8.9% |
| P5 | c.1129G>A (p.Val377Ile) ^#^ | c.442_447dupGCCTAC (p.Tyr149_Ser150insAlaTyr)^##^ | nd |
| P6 (Het) | c.1129G>A (p.Val377Ile) ^#^ | - | nd |
| P7 | c.1129G>A (p.Val377Ile) ^#^ | c.340_344del (p.Tyr114Ilefs*71) ^#^ | nd |
| Het1 | c.58C>A (p.His20Asn) ^#^ | - | nd |
| Het2 | c.1129G>A (p.Val377Ile) ^#^ | - | nd |
| Prnt1 (Het) | c.1129G>A (p.Val377Ile) ^#^ | - | nd |
| Prnt2 (Het) | c.340_344del (p.Tyr114Ilefs*71) ^#^ | - | nd |
